# A formative evaluation of the implementation of a medication safety data collection tool in English healthcare settings: A qualitative interview study using normalisation process theory

**DOI:** 10.1371/journal.pone.0192224

**Published:** 2018-02-28

**Authors:** Paryaneh Rostami, Darren M. Ashcroft, Mary P. Tully

**Affiliations:** 1 Division of Pharmacy and Optometry, School of Health Sciences, University of Manchester, Manchester Academic Health Sciences Centre (MAHSC), Manchester, United Kingdom; 2 National Institute for Health Research (NIHR), Greater Manchester Patient Safety Translational Research Centre, Greater Manchester, United Kingdom; 3 Manchester Health e-Research Centre, Division of Informatics, Imaging and Data sciences, School of Health Sciences, University of Manchester, Manchester, United Kingdom; SWEDEN

## Abstract

**Background:**

Reducing medication-related harm is a global priority; however, impetus for improvement is impeded as routine medication safety data are seldom available. Therefore, the Medication Safety Thermometer was developed within England’s National Health Service. This study aimed to explore the implementation of the tool into routine practice from users’ perspectives.

**Method:**

Fifteen semi-structured interviews were conducted with purposely sampled National Health Service staff from primary and secondary care settings. Interview data were analysed using an initial thematic analysis, and subsequent analysis using Normalisation Process Theory.

**Results:**

Secondary care staff understood that the Medication Safety Thermometer’s purpose was to measure medication safety and improvement. However, other uses were reported, such as pinpointing poor practice. Confusion about its purpose existed in primary care, despite further training, suggesting unsuitability of the tool. Decreased engagement was displayed by staff less involved with medication use, who displayed less ownership. Nonetheless, these advocates often lacked support from management and frontline levels, leading to an overall lack of engagement. Many participants reported efforts to drive scale-up of the use of the tool, for example, by securing funding, despite uncertainty around how to use data. Successful improvement was often at ward-level and went unrecognised within the wider organisation. There was mixed feedback regarding the value of the tool, often due to a perceived lack of *“capacity”*. However, participants demonstrated interest in learning how to use their data and unexpected applications of data were reported.

**Conclusion:**

Routine medication safety data collection is complex, but achievable and facilitates improvements. However, collected data must be analysed, understood and used for further work to achieve improvement, which often does not happen. The national roll-out of the tool has accelerated shared learning; however, a number of difficulties still exist, particularly in primary care settings, where a different approach is likely to be required.

## Background

One in ten patients are harmed by their healthcare and research indicates that 15% of this harm is medication related [1]. Large-scale epidemiological studies provide important insights into the problem, but are time-consuming and expensive to conduct. Voluntary incident reports have formed the mainstay of medication safety data within England’s National Health Service (NHS); however, whilst reports are vital for learning, they do not allow measurement and tracking of improvement over time. In order for healthcare organisations to know if they are reducing medication-related harm, medication safety must be routinely measured to provide a baseline and to track improvement. In order to address the lack of routine medication safety data, England’s NHS introduced an improvement tool called the “Medication Safety Thermometer” (MedsST) in 2013 [2] and developed it via a large-scale multi-disciplinary collaborative, facilitated by Haelo (an independent innovation and improvement centre hosted by Salford Royal NHS Foundation Trust, England).

The MedsST was developed based on a similar tool called the “original Safety Thermometer”, which routinely measures harms from pressure ulcers, falls, venous thromboembolism and urine infections in catheterised patients [3]. Following the national rollout of the original Safety Thermometer, four further Safety Thermometers were developed for maternity, mental health, children and young people and, the subject of this paper, the MedsST. The MedsST consists of three steps to measure individual errors, triggers of harm and actual harm related to medication safety. Details of the MedsST tool and how it has been designed and developed are described in a previous paper [2].

An evaluation of the original Safety Thermometer identified that it is possible to establish nationally used measurement systems to aid patient safety improvement, via large-scale collaborations. However, there were considerable challenges, for example changes to organisational policy at local and national levels, such as loss of funding for use of the intervention [4]. All NHS organisations were offered financial incentives to use the original Safety Thermometer through the Commissioning for Quality and Innovation (CQUIN) mechanism between April 2012 and March 2013. However, only regional and local financial incentives have been provided for organisations to use the MedsST. Only the Early Adopters (EA) of the MedsST (who joined the national programme during the alpha-testing phase, between January 2013 and March 2013) based in the Greater Manchester region, received a regional financial incentive for using the MedsST, between April 2013 and March 2014 [2]. The sole remaining EA organisation that was based outside of Greater Manchester, and Late Adopter (LA) organisations, who joined during the beta-testing phase or after (April 2013 onwards), have not received financial incentives unless they were agreed with their local commissioning groups [2]. The availability of financial incentives is an external contextual factor [5], and one of many differing contextual variables regarding how the MedsST has been implemented and used in comparison to the original Safety Thermometer. Another example of a contextual variable is the greater variety of healthcare staff that have been needed for development and use of the MedsST, due to the complexity of identifying medication-related harm [2], which has also been identified as a factor that influences successful implementation of a patient safety intervention [5].

Although literature exists evaluating the original Safety Thermometer and other large-scale patient safety systems, to date there is very little evidence evaluating patient safety measurement systems that focus specifically on measuring medication safety, which can be a more complex endeavour [2]. Using implementation theory to evaluate the implementation would also help understand the relevance to how a similar tool to the MedsST may be implemented in other healthcare settings.

Given the potential for medication safety measurement to help reduce medication related harm and, therefore, improve patient safety, and the lack of theory-based evidence relating to the implementation of routine medication safety measurement tools, particularly with a national focus, the aim of this study was to explore healthcare staff’s experiences of implementing the MedsST in England, using Implementation Theory[6].

## Methods

### Ethical considerations

University ethical approval was received from The University of Manchester Research Ethics Committee 3 on the 25th November 2015 (reference number 15479). Written consent was gained from participants prior to interviews.

### Design

As the research question is descriptive and concerns “how” the MedsST has been implemented, a qualitative approach was used to allow collection of data that could be used to gain an in-depth understanding of this phenomenon [7]. Interviews were used, which allowed rich data to be obtained by purposively recruiting a number of respondents that was small enough to permit in-depth qualitative analysis, but displayed wide diversity in perspective (i.e. a ‘maximum variation’ approach). Normalisation Process Theory (NPT) was used as an underlying concept for data analysis [8]. NPT is a theory of healthcare implementation and offers a structure for understanding practices that enable or constrain the integration of an intervention into routine care [9]. Reporting of this study is in line with the Consolidated criteria for reporting qualitative research (COREQ): and a COREQ checklist can be found in S1 File of the Supporting Information files[10].

### Sampling

Participants were recruited from various healthcare organisations in England, and included EA and LA organisations. Staff were purposely sampled to recruit staff who were leading the implementation of the MedsST (MedsST leads) and frontline users collecting data for the tool (MedsST users) based in healthcare organisations that had used the MedsST for at least 3 months consecutively, and that were based in England (where the MedsST has been exclusively developed and implemented). MedsST leads are appointed representatives of their organisations, who have been involved with the implementation of the MedsST at their organisations. MedsST leads are usually senior pharmacists; however, middle-grade pharmacists, pharmacy technicians and nurses may also be MedsST leads. MedsST users are usually nurses and pharmacists, but may also be pharmacy technicians, pre-registration pharmacists and clinical auditors. To determine sample size, the data saturation approach was used, where the researchers determine when the data collected from the interviews becomes redundant and it is estimated that the inclusion of more study subjects would add little to understanding of the study phenomenon [11].

MedsST leads were recruited by e-mail, using a database of existing contacts known to Haelo. This recruitment was followed up with snowball sampling, in which the MedsST leads were asked to forward the e-mail to MedsST users within their organisation. Some participants were known to the researcher through professional networks prior to the study.

### Data collection

Fifteen in-depth semi-structured interviews were conducted between December 2015 and September 2016 by PR, as part of her PhD project evaluating the use of the MedsST. The interview schedule (see S2 File) was based on three main topics drawn from the recommended national guidance about the MedsST (the most recent guidance is available from www.safetythermometer.nhs.uk) [12]; engagement with the purpose of the tool, data collection and the use of data by the organisation. To better understand what enables and constrains the implementation of a large-scale intervention in different settings, contexts were also considered by focussing on “how implementation processes differ between settings” [8], for example, questions about whether financial incentives were attached to use of the MedsST. The resulting interview schedule was piloted with a pharmacist before data collection began. New topics were added to the interview schedule, for subsequent interviews, as they arose in earlier interviews. Interviews were conducted in person, at the participants place of work, or by telephone, ranged in length from 32 to 99 minutes (average, 63 minutes), and were digitally recorded and transcribed *verbatim*. Field notes were also made and used to clarify the meaning of the interview data, such as the specialty of wards mentioned by the participants.

### Data analysis

The interview transcripts were imported into the qualitative data analysis management software QSR N-Vivo version 11.0. The first author (PR) conducted the data analysis, which was conducted in two stages, the co-authors (MPT and DMA) provided advice and input throughout the analysis process. The first stage involved an initial inductive thematic analysis of data, where data were coded, categorized and similar categories grouped into themes emerging from the data[13]. The constant comparative method was used, where the data analysed were constantly compared with earlier collected data, to form the categories and to explore variations in the data. The end result was the identification of 10 descriptive themes, shown in the second column of Table 1.The second stage involved a deductive theory-driven analysis of the data. Once the thematic analysis was complete, emergent themes were compared against existing implementation theories and frameworks [6]. Strong resonance was identified between the data, emergent themes and the NPT constructs, and it made sense to extend the analytical process by mapping the emergent themes onto the four NPT constructs [8, 9]. The constructs of NPT and the relevant themes from the thematic analysis are presented in Table 1, alongside working definitions of the NPT constructs for this specific study.

**Table 1:** Descriptive themes and their definitions.

Normalisation process theory construct	Descriptive theme	Definition
**Coherence: Understanding the purpose of the Medication Safety Thermometer and its data**	**Views on purpose**	Views on what the Medication Safety Thermometer is and how it should be used, as perceived by participants.
	**Operation**	How the Medication Safety Thermometer data were being collected, which represents the understanding of the wider teams within organisations regarding how the Medication Safety Thermometer should be used.
**Cognitive participation: Engagement with the Medication Safety Thermometer and its data**	**Organisational readiness**	The culture within organisations prior to using the tool, with respect to patient safety, auditing and quality improvement.
	**Ownership and engagement**	Ownership of medication safety overall and engagement with Medication Safety Thermometer data collection and use of data, for further improvement work.
	**Leadership and support**	Views on the impact of having individuals who lead implementation of the MedsST, and relevant support networks for those leading implementation and frontline users, at organisational, regional and national levels.
**Collective action: Activities undertaken to “normalise” Medication Safety Thermometer use into routine practice**	**Scaling up**	Actions taken, or planned, to scale up use of the tool.
	**Time and money**	Time and money as influences on collecting MedsST data and subsequent improvement work using the data
	**Education and training**	Details of associated training for staff involved with the use of the Medication Safety Thermometer
**Reflexive monitoring: Reviewing Medication Safety Thermometer use and embedding changes**	**Use of data**	How the data were actually used within organisations.
	**Reviewing and amending use of the tool**	Changes to the process of collecting Medication Safety Thermometer data to suit individual contexts. Including suggestions for the future.

The co-authors contributed to the analysis in discussion of data, themes and constructs, to ensure that all perspectives were covered. Quotes were chosen to best illustrate each theme and to display a range of varying opinions. Words in parenthesis have been added to quotes by the authors to clarify meaning, and ellipses (*…*) have been used to indicate the removal of unrelated text or information that may lead to identification of participants. Participant details are presented in Table 2.

## Results

Fifteen participants were recruited from ten organisations, including five EA organisations and five LA organisations (Table 2). The ten organisations were located across six different English counties. Participants consisted of eight MedsST leads and seven MedsST users. At the beginning of the study period (December 2015), staff from sixty-five organisations were eligible for the study according to the inclusion criteria, therefore 15% of eligible organisations were represented in the study. Participant roles included seven secondary care pharmacists, two primary care pharmacists, three secondary care nurses, one pre-registration pharmacist, one pharmacy technician and one clinical auditor. Despite considerable efforts, only two participants working in primary care were recruited, and both were pharmacists and MedsST leads. Openly accessible data online indicates that many primary care organisations had stopped using the MedsST prior to this study [12]. Implications of, and reasons for, primary care organisations stopping the use of the MedsST were explored during interviews with all participants, as certain secondary care organisations worked closely with associated primary care organisations.

**Table 2:** Participant details.

Organisation (EA/LA)	Participant	Implementation Role (Lead/User)	Profession	Setting Type
1 (LA)	1	Lead	Pharmacist (MSO*)	Secondary care
2 (LA)	2	Lead	Pharmacist (MSO)	Secondary care
	3	User	Clinical Auditor	Secondary care
3 (LA)	4	Lead	Pharmacist	Primary care
4 (LA)	5	User	Pharmacy Technician	Secondary care
	6	Lead	Pharmacist (MSO)	Secondary care
5 (LA)	7	User	Pharmacist	Secondary care
6 (EA)	8	Lead	Pharmacist (MSO)	Secondary care
	9	User	Nurse	Secondary care
7 (EA)	10	User	Nurse	Secondary care
	11	Lead	Pharmacist	Secondary care
8 (EA)	12	Lead	Pharmacist (MSO)	Secondary care
	13	User	Nurse	Secondary care
9 (EA)	14	Lead	Pharmacist (MSO)	Primary care
10 (EA)	15	User	Pre-registration Pharmacist	Secondary care

*MSO: Medication Safety Officer. **EA:** Early adopter (joined in the alpha-testing phase [January–March 2013]). **LA:** Late Adopter (joined in the beta-testing phase or after [April 2013 onwards]) [2].

### Factors influencing implementation of the MedsST

The findings are presented within the NPT framework, using study-specific definitions (see Table 1) and supported by illustrative quotes. The source of each quotation is indicated by participant number, profession and whether they are from an EA or LA organisation.

#### Coherence: Understanding the purpose of the MedsST and its data

Regarding the views on the purpose of the tool, the study analysis highlighted that a common understanding existed, concerning the rationale for measuring medication safety for learning and improvement. However, in order to have confidence to engage with the MedsST implementation, NHS staff required clarification on the operation of the tool, such as; how data should be collected and by whom, and available support networks that could be used to facilitate this. All participants were in agreement that the MedsST’s purpose was to aid improvement of medication safety by enabling organisations to quantify medication safety issues and providing a *“base-line”* (P10, Nurse, EA) to monitor improvement. Specifically, it was used to identify the most problematic hospital wards and work with them to improve medication safety.

Most organisations had previously used yearly audits to measure medication safety. Staff with training or passion for quality improvement truly understood the benefits of monthly medication safety data, as opposed to yearly data from the traditional medication safety audits collected by most organisations.

Only one organisation had collected monthly medication safety data prior to implementing the MedsST, using an internally developed tool. The MedsST lead from this organisation reported that their medication safety data tool had been replaced by the MedsST, because of its national focus, and the ability to use the learning they had gained from their previous tool to contribute to the MedsST’s development, as this MedsST lead had joined the steering group who were leading the development of the MedsST. The MedsST lead preferred the previous tool, due to greater data *“granularity”* (P12, Pharmacist, EA). However, the MedsST user in the same organisation preferred the MedsST over the previous tool, because it involved nurses, saved time and provided immediate feedback for wards.

*There was a clunky internal system before that resulted in our lead pharmacist having to plug away hours and hours and hours of data collation*, *and pulling together and feeding that back to us*. *That’s clearly not a robust way…(and the Medication Safety) Thermometer for our organisation is brilliant…it has slightly made our data collection better*, *I’d argue*. P13 (Nurse, EA)

Participants reported an initial lack of understanding about how data could be used for improvement, particularly in LA organisations, where data collection was initiated prior to gaining a full understanding of how it should be used. Some participants felt strongly that the MedsST should not be used for pin-pointing individuals and for staff to have *“the finger pointed at them*.*”* (P3, Clinical Auditor, LA). Nonetheless, one pharmacist (P2, LA) stated that MedsST data had been used for pin-pointing poor practice of nurses and subsequent performance management. Most nurses agreed with participant 2 that MedsST data should be used for monitoring performance of nurses and that practice cannot be completely *“blame-free”*, as certain *“stupid”* individuals could cause errors; however, they also believed that errors are actually caused by system problems that need to be addressed by supporting individuals. Although national online guidance specifically states that the tool has not been developed to blame individuals [12], the study analysis highlighted that cognitive dissonance may exist regarding whether staff believe medication errors are due to specific individuals, or system problems. Although it was stated that errors may be due to individuals, it was also stated that the errors individuals make are usually due to system problems.

*It can't be completely blame-free*, *because obviously some people are just stupid*. *But what you're not trying to do is beat them with a big stick*, *and look for trends*, *because usually when an error occurs it tends to be the system and not the person*. P9 (Nurse, EA)

Although more junior staff understood the purpose of the MedsST was to collect medication safety data, they were unsure about how data were actually used. Lack of coherence was reported to be a problem in primary care, where data were mainly collected by junior staff. One MedsST lead from primary care believed that junior staff may not understand the value of MedsST data and reported a culture of *“looking for mistakes”* (P4, Pharmacist, LA), rather than looking for harm (regardless of how it occurred), which may have led to missed learning opportunities.

*I wonder if the people there are quite junior…(and) are just doing what they are told by senior staff and…implementing it (MedsST)…not really thinking about the value of it…and thinking*, *'no that's not an error*, *we haven't done anything wrong'…I tried to explain…it’s not just about error*. P4 (Pharmacist, LA)

Although now based in primary care, Participant 4 had previous secondary care experience and quality improvement training. Therefore, they felt the culture of looking for errors, rather than harm, may be more predominant in the primary care settings, where there may not be organisational readiness for quality improvement tools, such as the MedsST.

#### Cognitive participation: Engagement with the MedsST and its data

In order for healthcare staff to engage with the MedST, they had to have ownership of their organisation’s medication safety, by being involved with for the medication process, for example, the clinical auditor (P3) was not involved with the medication process and showed less engagement as reported below. Furthermore, in organisations where staff displayed coherence with wider quality improvement projects, greater engagement and organisational readiness for implementing the MedsST was reported, and the use of the MedsST appeared to have “normalised” into routine practice more easily [14]. Involvement with the MedsST strengthened medication safety ownership and led to improvements in participants’ own practice.

*When I do come across a drug chart that hasn't been dated*, *I get quite frustrated because at the end of the day it is trust policy…with me having more involvement with this (the MedsST)*, *I am really aware of it now*. P5 (Pharmacy technician, LA)

Although difficulties with engaging ward senior management were reported, their involvement with data collection led to greater trust in and ownership of medication safety data.

*I think if I’m the ward sister and someone tells me…’You’ve got 10% dose omissions’ and they’ve done the audit*, *it’s useful…but if I’ve actually done it (collected MedsST data)*, *then I know that it was John in bed one and Barbara in bed three*. P13 (Nurse, EA)

It was evident from the data that strong support networks, internally and externally to organisations, seemed to be fundamental for impetus for improving medication safety. In particular the formation of the Medication Safety Officer (MSO) network in 2014 [15] provided a helpful support network for MedsST leads who were also the MSO for their organisation (an allocated member of staff to support local medication error reporting and learning [15]). Although external support did improve impetus for using data for improvement, this impetus was decreased if there was a lack of support internally, particularly from senior management (at ward and organisational management levels). Where senior management staff displayed a lack of ownership of the MedsST a knock-on effect could occur, where frontline staff would show less engagement. Furthermore, management staff had to be *“pro-active”* (P9, Nurse, EA) to ensure that ward staff were aware of the MedsST data, and for further improvement work to be conducted. Lack of engagement from management staff was associated with decreased awareness of the MedsST within organisations and, therefore, lack of use of data for improvement. It was reported that more junior staff, such as pre-registration pharmacists, collecting data displayed less engagement, feeling it was *“pushed”* onto them.

*As a group we feel like no-one wants to do it (collect data)*, *so they just pushed it towards the pre-reg (pre-registration pharmacists)*, *we won’t complain*, *we’ll just do it…(but) it would make more sense for a technician…to collect the data because they’re doing the meds rec (medicines reconciliation) every day*. *So they’re familiar with the chart*, *they’re familiar with the patient*. P15 (Pre-registration Pharmacist, EA)

As mentioned previously, the Clinical Auditor (P3, LA) who was interviewed was the only participant who was not directly involved with medication dispensing or administering in the rest of their work, and reported that they had been *“borrowed”* by the pharmacy department to help collect data. Therefore, they demonstrated a lack of ownership of medication safety improvement and did not feel inclined to view or act on the data they had collected. Furthermore, their lack of involvement in the medication process, led them to feeling like a *“lonely worker”* who was not part of *“the bigger picture”*. Some MedsST leads also felt unsupported in improving medication safety, if they had no one else in their teams.

*“In terms of governance and medication safety…I'm a bit of a one-man band…because…There isn't any other people (in my team)”* P1 (Pharmacist, LA)

This feeling of lack of support had detrimental effects on impetus for improving medication safety, some MedsST leads had overcome this lack of support by forming or joining external medication safety networks, where they could learn about how to use the MedsST and its data for improvement. The support networks were often developed by pro-active MedsST leads, and enabled wards, organisations and regions to share innovative methods of using data for improvement, in addition to learning how challenges with data collection could be overcome. In most organisations the MedsST lead was also an MSO, however, in one organisation the MSO was not involved with the MedsST and the role of MedsST lead had been offered to a pharmacist with personal interest in quality improvement. This participant had unsuccessfully attempted to contact other organisations to learn how they had implemented the MedsST in primary care settings. Without internal or external support networks, the MedsST role had proven burdensome for the pharmacist who felt unsupported and that they were not being *“listened to”* (P4, Pharmacist, LA) about how the MedsST data could be used for further improvement work.

*I am very aware that it shouldn’t really be my role…and I keep telling them that…“Yes I can implement this for you*, *I can tell you what the problems are etcetera*, *but you need to take it on”*. P4 (Pharmacist, LA)

Many organisations had not experienced the benefits of a multi-disciplinary approach, as described by other participants, as nurses were not involved with data collection. Nonetheless, even participants from organisations where only the pharmacy staff were collecting data, believed that measurement and improvement of medication safety required a multi-disciplinary approach. It was generally reported that senior nursing staff said they did support the use of the MedsST; however, they did not show this support and had often used *“lack of capacity”* (P6, Pharmacist, LA) as an excuse for not allowing nurses to be involved with MedsST data collection. Furthermore, it was highlighted that involvement of nursing staff at ward level was important for fostering multi-disciplinary ownership of medication safety.

*People see pharmacy…(and think) drugs*. *But…as nursing staff*, *we give them…They (pharmacists) don't actually administer them so there's no point (pharmacists) coming along thinking*, *‘I'll fill all this and I know what we're doing’*. *Yes*, *it's dead easy for you to fill it in*, *but then there's no ownership at ward level and then the nursing staff don't get to know what's going on*, *they don't actually see it*. *P*9 (Nurse, EA)

Generally, there was a lack of cognitive participation with Step 3, which involves a multi-disciplinary huddle to ascertain whether medication related harm has occurred, and the majority of organisations were not using it. Participants reported difficulties with gathering the pharmacist, nurse and doctor for a multi-disciplinary huddle. However, some organisations, despite facing similar staff and funding issues, reported that they had no issues with performing the huddle for Step 3 once people understood that it is a simple conversation between the nurse and junior doctor looking after the patient and the pharmacist collecting data.

*I think the term MDT (multi-disciplinary) huddle has made people think that that's some kind of super 'I need to get it sorted out by e-mail*, *I need to get the consultants in' and no*, *that isn't like that*, *and that is why I say*,*… ‘Look*, *just grab the junior doctor*, *grab the primary nurse and see if they agree*, *and then put it in the incident system*. *If that needs investigation then we might need to get the consultant in and we might need to get the ward manager in’*. P8 (Pharmacist, EA)

Mixed feelings were displayed about performing MDT huddles in primary care settings, the main benefit highlighted was the team-working between the pharmacist and a patient’s GP to review a patient’s safety in a novel way. However, there were practical difficulties with contacting the GP and making them aware of why they were being contacted.

*Initially they (the GPs) started off (saying) ‘what the hell are you ringing me about’…but when I explained it (the MedsST) to them*, *yes they could see that there was a value and a point to it*. *So yes*, *that was positive*. P4 (Pharmacist, LA)

Primary care NHS staff were generally supportive of the concept of the MedsST, but believed some questions were not applicable for their setting, and that using an alternative community version (version 16b) would not improve the issues faced. Of the four high risk drugs monitored using MedST, only insulin had triggered MDT huddles in primary care settings, leading to primary care organisations reviewing the suitability of these triggers. One primary care organisation had stopped using the MedsST for this reason, as reported by a participant from this organisation’s associated secondary care organisation.

*The uptake in community (primary care settings) is really poor*. *We've stopped doing it in community because…what was the point in doing it if we weren’t detecting anything? Therefore*, *we are clearly asking the wrong questions*. P8 (Pharmacist, EA)

#### Collective action: Actions taken to normalise use of the MedsST into routine practice

Many activities were reported to have been undertaken to scale up implementation of the MedsST within organisations, including increasing the number of wards data were collected from, and securing additional funding and staff for this. Non-financial incentives existed for staff for their involvement in collection of data, such as protected time for overseeing data collection and analysis, involvement with related research projects and attendance at medication safety meetings.

Local financial incentives for organisations to use the tool, had often been organised at the suggestion of the MedsST lead to the local Clinical Commissioning Groups (CCGs) who set financial targets for healthcare organisations in England [2]. The introduction of financial incentives were referred to by participants as a *“turning-point”* (P6, Pharmacist, LA), and had led to management staff giving the MedsST data “*greater respect”* (P8, Pharmacist, EA); and more senior staff involvement with data collection to ensure accuracy of data. Although it was difficult to involve senior staff, as they already had high work-loads, doing so had led to increased ownership and passion for improving medication safety and, therefore, supporting analysis of MedsST data.

*After…CQUIN (payments became available)*, *obviously we had reports to do*, *every quarter we had to say why we hadn't achieved*, *there were penalties and no financial gain and that's probably where the turning point was and because of that I started really looking at the data*. P6 (Pharmacist, LA)

Although financial incentives were reported to help drive data collection and scale-up of MedsST use, it was important to MedsST leads that they worked with local CCGs when introducing financial CQUIN payments, to ensure targets were realistic.

*The problem is CCGs get hold of something and they don't actually understand it*, *they just tell us to use it*. *So we have a potential CQUIN (financial incentive target) with one of the CCGs and my report has to actually…tell them how useful (the MedsST) is…and how much time it takes*. P4 (Pharmacist, LA)

Locally commissioned financial incentives appeared more successful than regionally commissioned financial incentives, and there was general agreement that national financial incentives should not be introduced. However, large-scale implementation nationally was described as a *“great thing”* (P2, Pharmacist, LA), but it introduced various complexities in different wards, organisations and regions, especially if they were still unsure of how to use the data for improvement. Participants discussed their organisation’s plans for scaling up use of the MedsST to all wards, which had been delayed multiple times, due to resistance from senior and ward level staff. Where organisations had successfully scaled up use of the MedsST to all wards, there was increased engagement of staff, and the MedsST had been embedded into routine practice to the point that it 'disappeared' from view (i.e., it was normalised) [14]. By contrast, in organisations where only some wards were collecting data, there was increased ward level resistance to implementation and by clinical champions (frontline users who take action to forward the implementation process) used personal friendships to overcome this.

*(The ward sister) was really against doing it and she used to rant and rave at me every time we talked about rolling it out… I said*, *'Look*, *I know (you don’t want to do it)*. *But*, *you’re going to do it for me anyway aren’t you?’*, *and she said*, *“Yes because you are my friend*, *but otherwise*, *no*, *I wouldn’t”*. P9 (Nurse, EA)

Although communication between MedsST leads with ward staff was occurring in most organisations to engage more wards to initiate use of the MedsST, communication was not occurring between senior organisational management, MedsST leads, ward level management regarding actually using the data collected, hindering the impetus for improvement using the collected MedsST data. For example, in organisations where data were analysed, the feedback was e-mailed to wards and not all frontline staff accessed e-mails. Additionally, for feedback to be acted upon, strong leadership was required from ward managers to create impetus for further improvement work.

*It has to be coming from a hierarchy saying*, *‘We need to develop something’*. *(If you) send out an e-mail…only ward managers read it and…not every ward manager is 100% proactive*. P9 (Nurse, EA)

Furthermore, there was a lack of education and training for healthcare staff about how to make best use of the data to inform quality improvement. Training was provided for data collection, but even this was problematic, with participants reporting a lack of funding and resources to train more staff. Most MedsST users had learnt how to collect data by shadowing other MedsST leads or users, and by using national guidance. One organisation had developed standard operating procedures in order to create a shared understanding between staff of how to collect consistent data, but these activities required extra funding. Many MedsST leads reported an inability to influence implementation to a greater extent, due to lack of funding, resources and support from senior levels who prioritised other areas of safety improvement. For example, it was reported that collecting data on electronic tablet devices (rather than paper-based) had halved data collection time; however, establishing the use of electronic tablets as a resource and gaining appropriate permissions presented challenges, and had taken 3 months in one organisation.

#### Reflexive monitoring: Reviewing use of the MedsST and embedding changes

To successfully embed the MedsST into routine practice, staff had to review their experiences of implementation and adapt the MedsST to suit local circumstances as necessary. Use of data included reviewing, analysing and trying to learn lessons from collected data. Although all participants were aware that they could access MedsST data via a dedicated website; however, very few had done so. Reasons for not viewing or using data included: technical difficulties (such as the website crashing), believing another department would view and act upon the data (such as the Quality Improvement department), time and resource constraints, communication issues and staff feeling ill-equipped or supported to analyse data.

Mixed feelings existed about the usefulness of data presentation on the dedicated website. For example, one MedsST user disliked the run charts used to display data and indicated preference for a written summary of change, which would require more management staff input.

*It would be nice to have a little summary to say*, *"This actually shows that 40% of patients have had their medicines" or something*. *Because… if you are not used to the…graph… (it’s) like "What's that line there for?" and "What does that mean there?"*. P9 (Nurse, EA)

Conversely, senior management staff preferred even more detailed displays, such as Statistical Process Control charts [16]. However, it is possible that frontline staff would not have the appropriate skills to interpret more detailed graphs, as they already reported difficulties understanding the simpler run charts, as indicated above. Staff who had quality improvement training or a personal interest, particularly from EA organisations, were more confident using MedsST data. EA organisations generally displayed better understanding and ownership of the MedsST, indicating that involvement of users with early development of an intervention can positively impact implementation of it.

Generally, the majority of participants did feel the run charts used to display data on the website were useful for visualising progress over time, and that the immediate access to these run charts was beneficial and time-saving, for example, in response to freedom of information requests.

*We had a freedom of information request about insulins*: *‘How (many) omitted doses have we had in the last quarter?’ Just like that*, *it was so brilliant*, *at a push of a button I could say*, *‘Oh yes*, *I know how many insulin doses were missed out of this proportion of these number of patients’*. P2 (Pharmacist, LA)

Where participants understood the data presentation, they successfully used run charts to monitor improvement, and identify patterns and trends. Furthermore, collecting data on a monthly basis had been vital for identifying and investigating certain trends, such as the impact of staffing shortages on medication safety in December every year, or the impact of system changes on medication safety (for example, the introduction of electronic prescribing). When investigating changes in data, input from ward staff was reported as vital.

*She (the ward sister) actually gave me reasons of why there was peaks in drug omissions*. *She said*, *'Around that time we had a lot of agency staff'…so that makes sense because agency staff obviously don't have that ownership (of good practice) that a permanent member of staff do*. P6 (Pharmacist, LA)

In addition, having routine medication safety data allowed users to demonstrate improvements at ward level, for example, for judicial inquiries.

*(We needed) to be able to prove … that things have actually improved since (an incident occurred)*. *…(So I thought) I’ll pull all the data off…so long as it looks good then we will send it*, *if it doesn't look good*, *let's not send it*. *But it did show that the ward (staff) were actually making strides to improve*. P9 (Nurse, EA)

MedsST data also allowed healthcare staff to identify specific areas of medication safety for further improvement work, for example, a medication omissions awareness project had been conducted by one organisation, as they had realised their rate of omissions was higher than the national average according to the MedsST data. Mixed feelings existed about using MedsST data for benchmarking and comparison between organisations, departments and wards. Some participants agreed it should happen; however, some participants felt that comparison data between organisations would be beneficial, but not between wards, as wards should be “*working together”* (P3, Clinical auditor, LA). However, other participants argued that comparison between different wards’ data did encourage wards working together by increased sharing of learning between wards. This culture of learning from each other was more apparent in EA organisations.

*Data on wards was being reviewed by other teams both internally and externally*, *and MedsST data was encouraging people to ask each other*, *“How did you get it right?”* P13 (Nurse, EA)

LA organisations had more recently started sharing lessons learnt from using the MedsST and related improvement projects, particularly between organisations. Although there are platforms, such as online forums, available for sharing information about how the MedsST has been used and received within organisations, some participants (particularly nurses) were not aware of them and felt there were communication issues between organisations.

*What goes on here might be brilliant*, *but unless someone is shouting about it from the rooftops*, *other wards might not be able to get to know about it*. P9 (Nurse, EA)

Communication between primary care MedsST leads was problematic and they reported a lack of awareness regarding how to contact their peers.

*I do think there might be potential (in primary care) but…I would like to speak to other community trusts to see how they are using it…and have looked at redesigning the triggers*. P4 (Pharmacist, LA)

## Discussion

This study has found that implementing medication safety measurement into routine practice is possible; however, collected medication safety data must be used for further local improvement in order to reduce medication-related harm, and this is not happening in most settings. A two-stage analysis approach was used, consisting of thematic analysis and an NPT framework, to better understand the barriers and facilitators experienced by English NHS staff who have implemented the MedsST into routine practice. The first stage of this two-stage approach, used in previous similar studies, helped to avoid forcing of data into predetermined conceptual categories and thus ensured our interpretation remained data-driven [17]. In the second stage, all themes from the first stage of analysis were mapped onto the NPT constructs, confirming the suitability of NPT as a suitable theory for evaluating patient safety interventions, such as the MedsST. However, as other researchers have found, there are challenges in differentiating the four NPT constructs [18]. The various elements of NPT interact in a complex adaptive system, where significant changes at a micro, meso or macro level manifest over time [8], highlighting the importance of factoring in context when evaluating interventions using NPT.

C*oherence* with routine medication safety data collection was achieved despite local context variation. Across all ten organisations there was agreement that the MedsST was useful for enabling routine medication safety measurement, and appropriate resources were in place to enable staff to *cognitively participate* with MedsST data collection, but not necessarily for using data for improvement. There were differences between settings regarding the *collective actions* that had occurred to upscale use of the MedsST, mainly due to the differences in resources, such as funding for extra staff. The study analysis confirmed findings from previous research that clinical champions are vital for successfully implementing and normalizing use of an intervention into routine practice, and that their key activities are to educate, advocate, build relationships and navigate boundaries [4, 19]. Our results suggest that clinical champions must not only *build* relationships, but utilise existing relationships to help implement improvement programmes. Through *reflexive monitoring* staff were able to evaluate how the MedsST can be improved and what changes were required. This ties into the methodology of MedsST development, which relied greatly on user feedback via Plan-Do-Study-Act cycles [20]. EA organisations were more able to influence improvement of the MedsST during development, which may explain the greater understanding of, and engagement with, further improvement work. The national focus was useful for organisations to learn from each other’s experiences of implementation; however, the implementation processes had to be adapted for each setting (ward, department or organisation). Each setting was an adaptive system that formed a dynamic environment(s) with different contexts [21]. Variation between these contexts contributed to determining intervention fidelity [8], therefore the varying contexts need to be considered and implementation processes adapted accordingly. The findings regarding the variations in how data are collected between settings, highlight the importance of being cautious when using MedsST data for comparison purposes, and understanding that contextual differences between settings may impact the data. However, the ability to share lessons learnt and view other settings’ data for learning purposes has been useful and this should be encouraged.

It was clear that in organisations where staff had greater knowledge and experience of quality improvement projects, due to a stronger quality and safety infrastructure [5], normalization of the use of the MedsST was simpler. A greater understanding of quality improvement concepts may lead to staff feeling more equipped to use improvement data, such as MedsST data, supporting previous calls for integration of quality improvement concepts into healthcare professionals training curricula [22]. One particular quality improvement concept that requires more awareness is the role of the system in the occurrence of errors. As previous research has highlighted, healthcare staff often may not understand the difference between a learning and a blame culture, and there is a lack of education provided enabling staff to differentiate between the two [23].

Lack of understanding about how data could be used for quality improvement, and who was responsible for reviewing and using data, existed and was due to communication issues. Clearer communication is required to make clear that all staff can review data and use it. This could help create a common culture and feeling of shared ownership of MedsST data, in turn increasing formative use of quality indicators [24].

Regular evaluation of interventions identifies barriers to normalising the use of an intervention as they occur and identifies whether improvement has occurred following changes. Technical issues resulting from misunderstanding definitions, highlighted the importance of communication about standardised definitions [2, 25]. Notably, users expressed that communication required major improvement, including awareness of support networks internally (within organisations) and externally (regionally and nationally), as the national focus is useless, if participants feel they have to *“shout from the rooftops”* about improvements they have made that could be generalised to other settings.

Participants agreed that there is a requirement for medication safety measurement in primary care settings, and most understood the need for routine data. However, participants felt that the medication classes used for the triggers of the MedsST [2] were not appropriate for most primary care settings (excluding community hospitals and intermediate care facilities), leading to discontinuation of the use of the tool by many primary care organisations.

One of the reasons for the difficulties in using the MedsST in primary care was the differences in infrastructure between primary and secondary care, and even between primary care sub-settings. For example, one pharmacist can cover a very large geographical area in the district care sub-setting, but there may be more than one pharmacist in a community hospital setting. Furthermore, within each type of primary care sub-setting, each organisation should adapt the MedsST to suit local contexts, and there is evidence of this occurring. For example, a recent study reported that a hospice in England was using the MedsST only for patients with more than ten medications [26], which saved resources yet helped to prevent harm in those patients considered most susceptible to medication-related harm.

Despite changes to the way the data were collected, the MedsST has still been deemed unsuitable for some primary care settings, where use of the tool has stopped. Participants felt this was due to the inappropriateness of the harms focussed on for primary care. It may be argued that the four high-risk medicines that the MedsST focuses, in particular, are inappropriate for most primary care settings. They were chosen based on all national incident reports, regardless of whether they were from primary or secondary care organisations [2], and previous research has found that most national medication-related reports are received from secondary care settings [27].

For the reasons highlighted above, it may be beneficial to redesign the community (primary care) version of the MedsST, and to combine use of the MedsST with the routine use of evidence-based initiatives currently used in primary care that are recommended by the National Institute of Clinical Excellence in the medicines optimisation guideline [28] in order to measure improvement over time. Many of these initiatives involve using electronic health records to identify the most prevalent potentially hazardous medication safety indicators that are specific to primary care [29–33]. Furthermore, sub-settings within primary care may require different sub-versions with specific measures. For example, measuring the number of patients who have been administered the wrong dose of medication, would be a relevant problem to measure for care homes [34] but probably not for district nursing settings (where nurses provide care to patients at their homes).

## Conclusion

Healthcare staff believed that standardised routine medication safety monitoring is a fundamental part of improving medication safety. However, medication safety can only be improved if data are analysed for learning purposes and lessons learnt are acted upon, which is not necessarily happening. This may be due to a lack of understanding about how data can be analysed and used. Developing quality improvement education for healthcare staff may help staff to become more confident with analysing data and using the findings for further improvement work. It may be beneficial to allocate further funding to improve medication safety, as organisations who have secured extra funding and staff for medication safety improvement reported the most improvement. However, an economic evaluation is required to ascertain this. Within organisations, there is a need to improve communication between multi-disciplinary teams from different wards, departments and management staff for more efficient use of the MedsST and its data for improvement. Greater communication between organisations is also required to spread best practices for implementation, and learn what works best for different contexts and save resources. Alternative approaches for measuring medication safety are required in different primary care settings, and participants perceived the current tool to be inappropriate for most primary care settings.

## Implications

There was great variation in participants’ awareness of whether their organisation had shown improvement of medication safety. Organisations could use the findings of this study to trigger further improvement work and investigation of collected MedsST data. For example, it may be useful to explore which organisations (or wards or settings) are showing improvement in medication safety, which may help us to understand the variation in practice and offer opportunity for learning and improvement. It is possible that variation exists in the prevalence of medication related harm between patient groups, settings, specialities and over time, and individual organizational data published online [29] demonstrates that, despite the constraints of using a tool that is relatively new, some organizations have improved [29]. This suggests that solutions to common problems may exist in the user community. Certain MedsST users, who are positive deviants, may have knowledge that can be generalized and, if the solutions have been generated within the MedsST user community, they may be more readily adopted in other organizations [35]. The standard methodology of the MedsST setting may be generalizable to other healthcare organisations internationally, but it is likely to require amendments to suit local contexts, for example, regarding who collects data. As alternative approaches for measuring medication safety are required in different primary care subsettings, the tool may also need to be amended to suit different primary care sub-settings, and further work is required to ascertain the potential of the MedsST for different primary care sub-settings.

## Strengths and limitations

To the best of our knowledge this is the first study to use explore views and experiences of staff using a medication safety measurement tool with a national focus.

Detailed insights were provided by a range of healthcare professionals holding a range of roles and levels of experience from a variety of healthcare settings and specialties. It may be argued that the work may have been strengthened by interviewing MedsST users in primary care settings; however, both MedsST lead participants from primary care settings had also acted as MedsST users and been involved with data collection within their organisations, enabling us to get an insight into how data collection actually occurs in primary care settings.

One of the main limitations of this study was that only staff who had volunteered were interviewed, indicating that they may be more proactive with medication safety improvement. However, it would be unethical to coerce participants and there were many negative opinions shared, suggesting that a range of views were represented.

As mentioned previously, no further data were collected after fifteen interviews because it appeared that data saturation regarding the implementation of the tool in hospital settings (where the tool is predominantly used) had occurred after 12 interviews. It is possible that data saturation regarding implementation of the tool in community settings did not occur; however, the most important finding in community settings was that the tool is not considered to be appropriate for these settings (excluding community hospitals). Although guidelines for sample size in qualitative research are varied and debatable, it is acknowledged that qualitative research typically involves the intensive study of a small group of people and tends to focus on depth rather than breadth [36].

Organisations that had used the tool for less than 3 months consecutively were excluded, in order to explore how MedsST use had been established and adopted over time. This may have led to the omission of some barriers that organisations faced, which resulted in their discontinued use of the MedsST. However, some participants were from organisations that had previously stopped using the MedsST and provided views about barriers to implementation.

The qualitative findings of this study highlight the importance of contextual factors in shaping how medication safety measurement can be implemented and normalised into practice, in different healthcare settings. These findings should help inform policymakers and organisations on how to optimise implementation of the MedsST into practice. Furthermore, the findings can also be used to develop and implement similar patient safety measurement tools internationally.

## Supporting information

S1 File COREQ checklist. A checklist to demonstrate that the reporting of this study is in line with the Consolidated criteria for reporting qualitative research (COREQ).
(DOCX)

S2 File Interview schedule. An approximate schedule that was used in the interviews.
(DOCX)
