# Supplementary material for: A formative evaluation of the implementation of a medication safety data collection tool in English healthcare settings: A qualitative interview study using normalisation process theory
Source: PLoS One. 2018 Feb 28;13(2):e0192224. doi: 10.1371/journal.pone.0192224 (PMC5830037; doi:10.1371/journal.pone.0192224)
Supplement: S1 File — A checklist to demonstrate that the reporting of this study is in line with the Consolidated criteria for reporting qualitative research (COREQ). (DOCX) [file pone.0192224.s001.docx]

**A formative evaluation of the implementation of a routine Medication Safety data collection tool used in English healthcare settings: a qualitative interview study using normalisation process theory**

**S1: COREQ checklist**

| **COREQ category** | **COREQ explanation** | **Response** | **Located** |
| --- | --- | --- | --- |
| Interviewer/facilitator | Which author/s conducted the interview or focus group? | Paryaneh Rostami (PR). Detailed in the methods, under ‘data collection’. | Page 6  (lines 12-13) |
| Credentials | What were the researcher's credentials? E.g. PhD, MD | MRPharmS. This is detailed in the information for the submitted manuscript on the title page. | Title page |
| Occupation | What was their occupation at the time of the study? | PhD student and pharmacist in community pharmacy. It has been stated in the funding information and competing interests that the researcher is a PhD student. | Submission information |
| Gender | Was the researcher male or female? | The main researcher (PR) is female. This has not been reported in the study as the issues are not gender specific. | N/A |
| Experience and training | What experience or training did the researcher have? | As a PhD student, PR has completed a Qualitative Research Methods module, and attended qualitative research training provided by the university of Manchester and is a practicing pharmacist.  Furthermore, the other members of the research team Darren M Ashcroft (DMA) who is an experienced researcher, and Mary P Tully (MPT) who is an experienced qualitative researcher. Both have undertaken and supervised many qualitative studies, and contributed to the design of the study protocol and interview schedule. Furthermore all authors input into analysis and writing of the manuscript, and MPT and DMA reviewed successive drafts of the paper. Authors’ contributions were provided at the submission stage. | Submission information  (re: authors’ contributions) |
| Relationship established | Was a relationship established prior to study commencement? | No personal relationships were established prior to study commencement. However, some participants were known to the researcher through professional networks. This is detailed in methods, under ‘sampling’. | Page 6  (lines 8-9) |
| Participant knowledge of the interviewer | What did the participants know about the researcher? e.g. personal goals, reasons for doing the research | At the start of each interview it was explained that the researcher was a PhD student. It was also explained that this study was part of her PhD project, which was funded by Haelo. This information is included in the interview schedule guide that is included as a supplementary file. This information was also provided on participants’ information sheets, which can be provided if requested by the editors. | File S2. |
| Interviewer characteristics | What characteristics were reported about the interviewer/facilitator? e.g. Bias, assumptions, reasons and interests in the research topic | It is mentioned in the manuscript, under ‘data collection’ that the data were collected for the main researcher’s PhD project. Furthermore, the researcher is interested in the topic because she is a pharmacist. However, she only works in community pharmacy settings and the MedsST is not used in these settings. Therefore, she did not have any pre-conceived assumptions before conducting interviews. | Page 6  (line 13) |
| Methodological orientation and theory | What methodological orientation was stated to underpin the study? e.g. grounded theory, discourse analysis, ethnography, phenomenology, content analysis | Interview guides were based on the recommendations for use of the intervention from national guidance. Analysis consisted of two stages; an initial general thematic analysis, followed by a subsequent analysis underpinned by Normalisation Process Theory. Details and reasons for this two-stage approach are included in both the ‘methods’ and ‘discussion’ sections. | Page 6  (lines 1-9) |
| Sampling | How were participants selected? e.g. purposive, convenience, consecutive, snowball | Participants were purposively sampled and this is stated in the method. Staff leading the implementation of the intervention (MedsST leads), from all eligible organisations, were invited to take part in the study. MedsST leads were then asked to forward the e-mail to frontline staff using the MedsST (MedsST users) within their organisation, using a snowball sampling approach. Details of the sampling have been provided in the ‘methods’ section under ‘sampling’. | Page 5  (lines 17-25)  &  Page 8  (lines 1-9) |
| Method of approach | How were participants approached? e.g. face-to-face, telephone, mail, email | MedsST leads were recruited using existing contacts known to Haelo, and asked to forward the e-mail to MedsST users within their organisation. This approach is described in ‘sampling’. | Page 6  (lines 6-9) |
| Sample size | How many participants were in the study? | There were 15 participants from 10 different organisations in the study. This has been described in the ‘results’ section, in terms of the number of participants and organisations that participated, as well as the proportion of eligible organisations that participated. | Page 9  (Table 2)  &  Page 10  (lines 2-8) |
| Non-participation | How many people refused to participate or dropped out? Reasons? | No-one dropped out of interviews. The poor participation rate from primary care staff is most likely due to primary care organisations stopping use of the tool. This has been described and discussed in the ‘results’ and ‘strengths and limitations’. | Page 10  (lines 8-13)  &  Page 31  (lines 1-4) |
| Setting of data collection | Where was the data collected? e.g. home, clinic, workplace | The interviews were conducted by telephone or in person (at the participant’s place of work). This is mentioned in the ‘data collection’ section. | Page 6  (lines 23-24) |
| Presence of non-participants | Was anyone else present besides the participants and researchers? | There were no non-participants present and this has therefore not been mentioned in the paper. | N/A |
| Description of sample | What are the important characteristics of the sample? e.g. demographic data, date | Interviews took place between December 2015-September 2016. This information is included in the methods. In addition, it is stated that organisations from six different counties in England took part to highlight the variation; however, these counties have not been named to preserve anonymity. | Page 6  (lines 12-13) |
| Interview guide | Were questions, prompts, guides provided by the authors? Was it pilot tested? | An interview guide was used, which has been included as Supplementary file 2 (S2). The interview guide was pilot tested with a pharmacist. | Page 6 (line 21) |
| Repeat interviews | Were repeat interviews carried out? If yes, how many? | No repeat interviews were conducted. | N/A |
| Audio/visual recording | Did the research use audio or visual recording to collect the data? | The interviews were digitally recorded and this is noted in the methods. | Page 6  (lines 24-25) |
| Field notes | Were field notes made during and/or after the interview or focus group? | Field notes were made and used to clarify interview data meanings. This is detailed in the data collection section | Page 6  (lines 25-26) |
| Duration | What was the duration of the interviews or focus group? | Interviews ranged in length from 32 to 99 minutes (average length, 63 minutes). This information is in the data collection section. | Page 6  (lines 24-25) |
| Data saturation | Was data saturation discussed? | Data saturation regarding implementation of the tool was reached as no new themes emerged after the 13^th^ interview. Data saturation has been discussed in the ‘sampling’ and ‘strengths and limitations’ sections. | Page 6  (lines 1-4)  Page 30  (21-25) |
| Transcripts returned | Were transcripts returned to participants for comment and/or correction? | No, due to the busy nature of healthcare professionals’ jobs, and the high-quality of recordings, it was felt that this would not be a useful exercise. | N/A |
| Number of data coders | How many data coders coded the data? | The main researcher (PR) coded data, with input and guidance from the rest of the research team during monthly team meetings. This is detailed in the data analysis section of the method and | Page 7  (lines 4-7) |
| Description of the coding tree | Did authors provide a description of the coding tree? | No, but this can be provided to the editors if required. Themes from the thematic analysis, and how they map onto Normalisation Process Theory constructs can be found in Table 1. | Page 7-8  (Table 1) |
| Derivation of themes | Were themes identified in advance or derived from the data? | Themes were derived from the data and mapped onto NPT constructs. This is explained and referenced in the data analysis section of the method. | Page 7  (lines 2-18) |
| Software | What software, if applicable, was used to manage the data? | Qualitative data analysis management software QSR N-Vivo version 11.0 was used, and this is detailed in the data collection section. | Page 7  (lines 3-4) |
| Participant checking | Did participants provide feedback on the findings? | No, due to the difficulties in arranging meetings with participants, because of the busy nature of healthcare professionals jobs and the time limitations of this study (which is part of a wider PhD project with a timeline) this was not possible. | N/A |
| Quotations presented | Were participant quotations presented to illustrate the themes / findings? Was each quotation identified? e.g. participant number | Yes and they have been identified by participant numbers, profession and whether they are from an Early or Late Adopter organisation (depending on which stage of testing of they adopted the intervention). | Results section (pages 19-24) |
| Data and findings consistent | Was there consistency between the data presented and the findings? | Yes, data have been provided as evidence for the findings, and inferences drawn from the data were described in the discussion. | Results section (pages 19-24)  &  Discussion  (pages  (23-30) |
| Clarity of major themes | Were major themes clearly presented in the findings? | Yes, the manuscript does not label the themes as major and minor. However, the major themes found were the NPT constructs which can be found in Table and are discussed in detail in the results section and discussion. | Page 7  (Table 1) |
| Clarity of minor themes | Is there a description of diverse cases or discussion of minor themes? | Minor themes have been mapped onto major themes (see Table 1) and have been discussed within each major theme and the results section. | Page 7  (Table 1) |
