# Supplementary material for: A formative evaluation of the implementation of a medication safety data collection tool in English healthcare settings: A qualitative interview study using normalisation process theory
Source: PLoS One. 2018 Feb 28;13(2):e0192224. doi: 10.1371/journal.pone.0192224 (PMC5830037; doi:10.1371/journal.pone.0192224)
Supplement: S2 File — An approximate schedule that was used in the interviews. (DOCX) [file pone.0192224.s002.docx]

## S2: Interview Schedule

## Interview schedule

This is the general list of topics discussed, which was adapted as necessary for each interview, to collect relevant data about the use of the Medication Safety Thermometer (MST) within interviewee’s organisations.

**Introduction**

This study is an exploratory study, and is the first study of my PhD project. The research is funded by Haelo and your participation is entirely voluntary. It aims to get an idea of your experiences and perceptions of using the MST in your organisation. There are no right or wrong answers to the following questions as I am interested in **your** experiences and perceptions of using the MST.

**Background details of participant:**

What is your organisation name and type of organisation (if not previously established)?

What is your present job title and how long have you been in this position?

**Uncderstanding and Engagement (Coherence/ collective action)**

In your view, what is the purpose of the Medication Safety Thermometer?

Is it being used for this purpose?

Was any data on medication error collected by your organisation prior to using the MST?

If yes, how was this done?

Why did you decide to use the MST? Was there financial incentives attached to use?

What training did staff have prior to being involved with the MST data collection?

Online or in person?

How long did this last?

**What is happening – Data collection (Cognitive participation/ collective action)**

Can you talk me through the process of collecting the data in your organisation? (for those who do the data collection themselves)

When is the Medication Safety Thermometer used? (recommended day? Does the day change?)

Who collects the data?

Who else is involved with data collection?

How is data recorded and submitted (paper/PC/iPad/both)?

What data sources do you use?

Do you use any unique identifiers for each patient to enable you to go back?

Does anyone check the data before it is submitted?

Do you use step 3, if so how long have you used it for.

Who decided who would collect the data?

What are the challenges associated with data collection?

How can they be overcome?

Do you use electronic prescribing at your hospital?

If not, are you planning to?

How does/ will it impact data collection?

How does you hospital operate over the weekend?

**Use of data (collective action/ reflexive monitoring)**

Do you think the data is understandable?

How do you use the data at your organisation?

- How did you expect to use the data and what did you think it would show? Did this happen?

Do you use the data for improvement?

Are you doing improvement work associated with the measures?

Where is the data discussed/reported to/displayed?

What is the impact of the data collected on patient safety in your organisation?

Do you trust the data from the MST?

Why?

What about other data sources other than MST data?

How do you use other medication safety data sources?

**Improvements (reflexive monitoring/coherence)**

How would you improve any step of the Medication Safety Thermometer?

What would you add? What would you remove?

Has the data given you information that has been uprising or that has told you things you did not already know?

Has participation changed your organisations approach to harm free care or medication safety improvement?

What do you think are the main benefits, if any, of using the MST?

How do you feel about using an unfinished tool?

Some organisation have stopped and started using the tool?

Do you think your organisation would have used the tool without associated financial targets? (if applicable)

What would happen if you left?

**Anything not covered?**

Is there anything that we have not covered in the interview that you would like to discuss?
